# Lipid accumulation product is an effective predictor of metabolic syndrome in non-obese women with polycystic ovary syndrome

**DOI:** 10.3389/fendo.2023.1279978

**Published:** 2024-01-10

**Authors:** Wenju Han, Meiwei Zhang, Haiyan Wang, Yitian Yang, Lei Wang

**Affiliations:** ^1^ Department of Reproductive Center, Dalian Women and Children’s Medical Group, Dalian, China; ^2^ School of Public Health, China Medical University, Shenyang, China

**Keywords:** polycystic ovary syndrome, metabolic syndrome, lipid accumulation product, nonobese, ROC curve analysis

## Abstract

**Objective:**

To explore the correlation of lipid accumulation product (LAP) with metabolic syndrome (MS) and to assess the predictive value of LAP for MS risk in polycystic ovary syndrome (PCOS) with different body mass index (BMI).

**Methods:**

A total of 242 PCOS patients and 150 controls were recruited and divided into normal-weight, overweight, and obese groups, then further divided into MS and without MS subgroups. Clinical and anthropometric variables and laboratory results were recorded. LAP was calculated from waist circumference (WC) and triglyceride using sex-specific formulae. Logistic regression analysis and receiver operating characteristic (ROC) curve were applied to determine and analyze the predictive value of LAP for MS.

**Results:**

The prevalence of MS among PCOS patients was 45.04%, which was significantly higher than that of the controls (10%). Stratified by BMI, the incidence of MS in the normal-weight, overweight, and obese PCOS groups were 15.58%, 41.43%, and 71.58%, respectively. Logistic regression analysis indicated that LAP was an independent risk factor for MS in both normal-weight and overweight groups; however, the results were not significant in the obese group. ROC curve analysis showed that LAP had an outstanding discrimination index for MS in normal-weight (AUC=0.960, cut-off value=42.5) and overweight (AUC=0.937, cut-off value=47.93) PCOS patients, with a sensitivity of 0.917/0.931 (normal-weight/overweight) and a specificity of 0.969/0.854 (normal-weight/overweight), respectively.

**Conclusion:**

Normal-weight and overweight PCOS patients also have a fairly high incidence of MS and should receive as much attention as obese patients. Compared to applying multiple clinical indicators, LAP is more convenient and facilitates acquiring early and accurate diagnoses of MS among non-obese PCOS patients using fewer MS markers.

## Introduction

Polycystic ovary syndrome (PCOS) is one of the most widespread endocrinopathies, affecting nearly 5.6% of Chinese reproductive-aged women (1). The incidence has been reported as up to 20% in some populations (2). Besides reproductive and obstetric problems, it is also closely related to the long-term metabolic consequences, including insulin resistance (IR), type 2 diabetes mellitus (DM) (3), metabolic syndrome (MS) (4), and cardiovascular disease (CVD) (5).

Metabolic syndrome (MS) is a series of metabolic disorders, including excessive accumulation of abdominal fat, hypertension, dyslipidemia, and hyperglycemia, and shares a lot of similarities with PCOS in terms of metabolism (6). PCOS women have been found to be at increased risk of metabolic syndrome compared to controls, with the greatest increase in the Americas (OR 5.21), and a significant increase in Australia and New Zealand (OR 3.55), Asia (OR 3.47), and Europe (OR 2.68) (7). Another study has shown that women with PCOS have an 11-fold increased risk (47.3% vs. 4.3%) of developing MS compared to their age-matched controls (8). Considering the long-term health risks associated with these conditions, early identification and intervention in these individuals with MS is necessary to prevent them from developing adverse consequences.

The diagnostic criteria for MS require the consideration of multiple anthropometric and clinical parameters. A series of studies focused on simple clinical indexes for early screening of MS in general populations, as well as for PCOS patients (9–14). Among them, lipid accumulation product (LAP), which is based on the assessment of waist circumference (WC) and serum triglycerides (TG), has been recommended as a credible indicator of the risk of IR in adult Americans (15), MS in adults and the elderly population (9, 10), and MS in Chinese children and adolescents (16). It is also proven to be more effective in predicting MS compared to the other seven indices, namely, Chinese visceral adiposity index (CVAI), waist circumference-triglyceride index (WTI), visceral adiposity index (VAI), triglyceride-glucose index (TyG), TG/HDL-C ratio, WC, and BMI in Chinese women with PCOS (13). However, studies were mostly performed in the PCOS population as a whole. There have been no reports of the value of LAP for predicting MS in PCOS individuals according to different BMIs. The present study aims to investigate the prevalence of MS in northeastern Chinese women with PCOS and to assess the predictive effect of LAP on MS in PCOS patients stratified by BMI.

## Materials and methods

### Subjects

This retrospective cross-sectional study including 242 patients with PCOS and 150 control women (aged 20-40 years) from north-east China was conducted in the outpatient department of the reproductive center of Dalian Women and Children’s Medical Group from June 2021 to October 2022. Informed written consent was obtained from all enrolled participants.

Each patient with PCOS met the diagnostic criteria for PCOS according to the revised 2003 Rotterdam criteria (17), i.e. the presence of at least two of the following three characteristics: (i) oligo- and/or anovulation (less than 8 menstrual cycles per year or menstrual cycles more than 35 days in length), (ii) clinical hyperandrogenism (acne or modified Ferriman-Gallwey scores ≥ 8) or biochemical hyperandrogenism (serum total testosterone level ≥2.6 nmol/L, free testosterone level ≥6.0 pg/mL), (iii) polycystic ovary morphology, which was confirmed if there were 12 or more follicles measuring 2-9mm in diameter in each ovary by ultrasonic examination.

The control participants consisted of healthy volunteers and infertile women due to fallopian obstruction or reason’s specific to their husband. All of them were clinically healthy, had regular menstrual cycles, showed neither clinical nor biochemical signs of hyperandrogenism, and exhibited normal ovarian morphology under ultrasonic examination.

The exclusion criteria were as follows: Cushing’s syndrome, congenital adrenal hyperplasia, androgen-secreting tumors, premature ovarian failure, ovarian surgery, endometriosis, thyroid dysfunction, hyperprolactinemia, clinically evident acute or chronic diseases, or sex hormone medication within three months.

### Anthropometric and biochemical measurements

Clinical and anthropometric variables were measured in all subjects. Body weight in light clothing and barefoot height were obtained and body mass index (BMI, kg/m^2^) was calculated. Waist circumference (WC) was measured as the smallest circumference at the level of the umbilicus. Systolic/diastolic blood pressure (SBP/DBP) was measured on the right arm after a 10-minute rest. Blood samples were obtained in the morning after overnight fasting between days 2–4 of menstruation/withdrawal bleeding after progestogen administration, and tested for different indexes by laboratory assays.

BMI was calculated using the following formulae: weight (kg)/height^2^ (m^2^). According to the Expert Consensus on Obesity Prevention and Treatment in China, normal-weight, overweight, and obese were identified as 18<BMI<24kg/m^2^, 24≤BMI<28kg/m^2^, and BMI≥28kg/m^2^, respectively (18). Insulin sensitivity was estimated by the homeostasis model assessment (HOMA-IR): [fasting insulin in µIU/ml ◊fasting plasma glucose (FPG) in mmol/L]/22.5. IR was considered as HOMA-IR ≥2.3 (19). Lipid accumulation product (LAP) was calculated from WC and triglyceride(TG) using the following formulae: LAP for women = (WC in cm -58) ◊TG in mmol/L (20).

### Metabolic syndrome

Metabolic syndrome (MS) was defined according to the National Cholesterol Education Program-Adult Treatment Panel (NCEP-ATP) III criteria modified for the Asian population. Diagnosis was made when there were three or more of the following risk factors: (і) central obesity with WC ≥80 cm in women; (ii) SBP≥130 mmHg or DBP≥85 mmHg and/or having been diagnosed as hypertensive and undergone treatment; (iii) fasting TG≥1.7 mmol/L; (iv) fasting HDL-C ≤1.29 mmol/L; (v) fasting glucose≥5.6 mmol/L and/or having been diagnosed as diabetic and undergone treatment (21).

### Statistical analysis

All statistical analysis was performed using SPSS version 21.0 (SPSS Inc., Chicago, IL, USA). Data were presented as mean ± standard deviation (SD) or median (interquartile range) as appropriate. The Kolmogorov-Smirnov test was used to test the normality of the distribution of all continuous variables. The comparisons between two normally distributed independent samples were performed using the Student’s *t*-test. The Mann-Whitney *U*-test was used for non-normally distributed variables. Differences in percentages were evaluated by χ^2^ tests between PCOS and control subjects. Relationships between LAP and other variables were tested by Pearson correlation analysis. Logistic regression analysis was used to investigate potential factors associated with MS. Receiver operating characteristic (ROC) curves analysis was used to assess the importance of the LAP in predicting MS. For all analyses, a *P*-value of <0.05 was considered statistically significant.

## Results

### Prevalence of MS and its components in patients with PCOS and control participants stratified by BMI

**Table 1** shows the prevalence of MS and each of the abnormal items of MS in PCOS and control subjects. In the PCOS group, the proportions of normal weight, overweight, and obesity were 31.8% (77/242), 28.93% (70/242), and 39.26% (95/242). The values for the control group were 64% (96/150), 32% (48/150), and 4% (6/150). PCOS patients had a higher prevalence of obesity compared to controls (39.26% versus 4%, *P*=0.000). Based on the NCEP-ATP III criteria, the total incidence of MS in northeastern Chinese women with PCOS reached up to 45.04% (109/242), almost 4.5 times as high as that in the controls, which was 10% (15/150). Stratified by BMI, the frequency of MS was significantly higher in the normal-weight PCOS group when compared with the control (15.58% versus 1.04%, *P*=0.000). For the overweight group, the frequency of MS in PCOS patients was 41.43%, higher than that of the control (27.08%); however, the difference was not statistically significant (*P* =0.110). The MS rate in the obese PCOS group was the highest (71.58%), while the obese control was only 16.67% (1/6). Due to the small number of obese controls, analysis was not performed between obese PCOS and controls. The total prevalence of each metabolic abnormality in patients with PCOS was significantly higher than that in the control group. After being stratified by BMI, compared with controls, normal-weight PCOS showed significantly higher rates of dyslipidemia, involving elevated TG (28.57% versus 4.17%, *P*=0.000) and decreased HDL-C levels (45.45% versus 25%, *P*= 0.005). Overweight PCOS had significantly higher rates of elevated blood pressure when compared with overweight control (21.43% versus 4.17%, *P*=0.009).

**Table 1:** Prevalence of components of MS in PCOS and control participants stratified by BMI.

items	Total			Normal weight			Overweight			Obese		
	PCOS	Control	*P*	PCOS	Control	*P*	PCOS	Control	*P*	PCOS	Control	*P*
N (%)	242	150	**-**	77(31.82)	96(64)	**0.000**	70(28.93)	48(32)	0.519	95(39.26)	6(4)	**0.000**
MS	109(45.04)	15(10)	**0.000**	12(15.58)	1(1.04)	**0.000**	29(41.43)	13(27.08)	0.110	68(71.58)	1(16.67)	**-**
WC≥80 cm	182(75.21)	74(49.33)	**0.000**	25(32.47)	24(25)	0.279	62(88.57)	44(91.67)	0.585	95(100)	6(100)	**-**
TG≥1.7 mmol/L	111(45.87)	18(12)	**0.000**	22(28.57)	4(4.17)	**0.000**	31(44.29)	13(27.08)	0.058	58(61.05)	1(16.67)	**-**
HDL-C<1.29 mmol/L	165(68.18)	57(38)	**0.000**	35(45.45)	24(25)	**0.005**	53(75.71)	29(60.42)	0.076	77(81.05)	4(66.67)	–
FPG≥5.6 mmol/L	50(20.66)	15(10)	**0.006**	10(12.99)	6(6.25)	0.128	13(18.57)	8(16.67)	0.790	27(28.42)	1(16.67)	–
BP≥130/85 mmHg	44(18.18)	7(4.67)	**0.000**	1(1.3)	4(4.17)	0.263	15(21.43)	2(4.17)	**0.009**	28(29.47)	1(16.67)	–

WC, Waist circumference; TG, triglyceride; HDL-C, high-density lipoprotein-cholesterol; FPG, fasting plasma glucose; BP, blood pressure.

Differences in percentages between PCOS and control groups were evaluated by χ2 tests. The bold P-value represents a statistical significance.

### General characteristics of the PCOS participants with or without MS stratified by BMI

Comparing the participants with MS-PCOS and without MS-PCOS, LAP (*P*=0.000), HOMA-IR (*P*=0.000), HDL-C (*P*=0.037), ALT (*P*=0.029), and UA/Cr ratio (*P*=0.003) showed significant differences in the normal-weight group. In the overweight group, LAP (*P*=0.000), DBP (*P*=0.037), HOMA-IR (*P*=0.000), TC (*P*=0.007), HDL-C (*P*=0.005), LDL-C (*P*=0.027), ALT (*P*=0.000), and AST (*P*=0.000) showed significant differences. Significant differences were observed in the mean LAP (*P*=0.001), SBP (*P*=0.000), DBP (*P*=0.003), HOMA-IR (*P*=0.000), ALT (*P*=0.036), and UA/Cr ratio (*P*=0.000) in the obese group (**Table 2**).

**Table 2:** The anthropometric and biochemical characteristics of the PCOS participants stratified by BMI.

items	Normal-weight-PCOS (n=77)			Overweight-PCOS (n=70)			Obese-PCOS (n=95)		
	MS (12)	non-MS (65)	*P*	MS (29)	non-MS (41)	*P*	MS (68)	non-MS (27)	*P*
Age(years)	32.92 ± 3.65	31.45 ± 2.95	0.131	31.59 ± 3.57	31.76 ± 3.57	0.845	31.37 ± 3.98	30.85 ± 3.51	0.558
BMI(kg/m^2^)	22.59 ± 1.12	22.10 ± 1.21	0.190	25.93 ± 1.05	25.80 ± 1.14	0.648	30.99 ± 2.28	30.54 ± 1.98	0.367
LAP	54.74(45.54,62.74)	19.80(12.87,27.57)	**0.000**	71.74(54.0,94.48)	29.7(19.36,40.5)	**0.000**	80.55(59.22,97.54)	53.0(31.53,80.69)	**0.001**
SBP(mmHg)	113.75 ± 7.21	111.17 ± 8.87	0.345	124.59 ± 11.63	120.44 ± 6.78	0.092	127.84 ± 11.86	119.44 ± 5.37	**0.000**
DBP(mmHg)	71.75 ± 7.84	70.54 ± 7.05	0.592	80.79 ± 8.00	77.24 ± 5.96	**0.037**	82.71 ± 8.69	78.52 ± 4.62	**0.003**
HOMA-IR	4.39(3.01,7.51)	2.12(1.60,3.23)	**0.000**	4.72(2.93,6.99)	3.2(2.03,4.04)	**0.000**	6.95(5.24,8.75)	3.97(2.75,5.30)	**0.000**
TT(ng/ml)	0.41 ± 0.25	0.29 ± 0.18	0.055	0.36 ± 0.16	0.38 ± 0.19	0.578	0.42 ± 0.21	0.37 ± 0.23	0.361
TC(mmol/L)	5.12 ± 0.73	4.75 ± 0.90	0.193	5.32 ± 0.98	4.68 ± 0.93	**0.007**	5.09 ± 0.83	4.86 ± 0.96	0.244
HDL-C(mmol/L)	1.18 ± 0.35	1.40 ± 0.33	**0.037**	1.10 ± 0.17	1.27 ± 0.31	**0.005**	1.08 ± 0.46	1.26 ± 0.27	0.059
LDL-C(mmol/L)	3.13 ± 0.68	2.83 ± 0.66	0.149	3.22 ± 0.83	2.77 ± 0.79	**0.027**	3.14 ± 0.74	2.89 ± 0.73	0.138
ALT(U/L)	22.5(15.25,47.5)	15.0(11.0,24.0)	**0.029**	28.0(19.0,36.0)	14.0(11.0,21.0)	**0.000**	35.0(23.0,51.0)	26.0(14.75(39.5)	**0.036**
AST(U/L)	18.5(15.0,29.75)	17.0(15.0,22.0)	0.299	21.5(18.0,27.5)	16.0(15.0,20.0)	**0.000**	24.0(17.5,30.5)	20.0(15.75,26.5)	0.129
LDH(U/L)	158.08 ± 39.10	167.50 ± 29.15	0.346	169.21 ± 35.44	164.0 ± 24.56	0.483	182.39 ± 27.79	178.85 ± 27.42	0.583
UA/Cr ratio	7.12 ± 1.69	5.76 ± 1.37	**0.003**	6.79 ± 1.92	6.08 ± 1.65	0.100	7.64 ± 1.49	6.36 ± 1.19	**0.000**

BMI, body mass index; LAP, lipid accumulation product; SBP, systolic blood pressure; DBP, diastolic blood pressure; HOMA-IR, homeostasis model assessment of insulin resistance; TT, total testosterone; TC, total cholesterol; HDL-C, high-density lipoprotein-cholesterol; LDL-C, low-density lipoprotein-cholesterol; ALT, alanine transferase; AST, aspartate transferase; UA/Cr ratio, uric acid/creatinine ratio. The bold P-value represents a statistical significance.

### Correlations between LAP and other parameters among PCOS patients participants stratified by BMI

The correlation analysis results between LAP and other parameters among PCOS patients are shown in **Table 3**. In the normal-weight group, age, BMI, SBP, DBP, HOMA-IR, ALT, and UA/Cr ratio were significantly positively correlated with LAP. In the overweight group, there were significant positive correlations between LAP and SBP, DBP, HOMA-IR, TC, ALT, and AST. However, in the obese group, only BMI and UA/Cr ratio were positively correlated with LAP. Significant negative correlations were found between HDL-C and LAP in the non-obese PCOS groups (normal and overweight).

**Table 3:** Correlations between LAP and other parameters among PCOS patients stratified by BMI.

items	Normal-weight-PCOS		Overweight-PCOS		Obese-PCOS	
	R	*P*	R	*P*	R	*P*
Age(years)	0.251	**0.028**	0.044	0.718	0.086	0.408
BMI(kg/m^2^)	0.262	**0.021**	0.139	0.250	0.382	**0.000**
SBP(mmHg)	0.386	**0.007**	0.416	**0.000**	0.025	0.809
DBP(mmHg)	0.484	**0.001**	0.441	**0.000**	0.103	0.319
HOMA-IR	0.369	**0.001**	0.433	**0.000**	0.177	0.085
TT(ng/ml)	0.156	0.064	0.105	0.093	0.081	0.398
TC(mmol/L)	0.200	0.083	0.279	**0.019**	0.195	0.058
HDL-C(mmol/L)	-0.381	**0.001**	-0.348	**0.003**	-0.113	0.277
LDL-C(mmol/L)	0.222	0.054	0.218	0.069	0.124	0.233
ALT(U/L)	0.335	**0.004**	0.256	**0.037**	0.092	0.384
AST(U/L)	0.204	0.083	0.257	**0.036**	0.072	0.499
LDH(U/L)	0.072	0.565	0.212	0.088	0.036	0.737
UA/Cr ratio	0.292	**0.010**	0.206	0.086	0.227	**0.027**

BMI, body mass index; SBP, systolic blood pressure; DBP, diastolic blood pressure; HOMA-IR, homeostasis model assessment of insulin resistance; TT, total testosterone; TC, total cholesterol; HDL-C, high-density lipoprotein-cholesterol; LDL-C, low-density lipoprotein-cholesterol; ALT, alanine transferase; AST, aspartate transferase; UA/Cr ratio, uric acid/creatinine ratio. The bold P-value represents a statistical significance.

### Logistic regression analyses of the major confounding factors for MS

The association between indices and the risk of MS in PCOS patients is presented in **Table 4**. After the multivariable logistic regression, LAP and HOMA-IR were still independent risk factors for MS in the normal-weight group (LAP: OR=1.21, 95%CI=1.06-1.37, *P*=0.004; HOMA-IR: OR=3.81, 95%CI=1.12-12.99, *P*=0.033). However, for the overweight group, only LAP remains an independent risk factor for MS (OR=1.10, 95%CI=1.037-1.162, *P*=0.001). In the obese group, HOMA-IR and UA/Cr ratio, instead of LAP, were independent risk factors for MS (HOMA-IR: OR=1.46, 95%CI=1.1-1.947, *P*=0.009; UA/Cr ratio: OR=2.09, 95%CI=1.251-3.479, *P*=0.005).

**Table 4:** Logistic regression analysis for MS in PCOS patients stratified by BMI.

items	OR (95%CI)	*P*
Normal-weight		
LAP	1.21(1.06-1.37)	**0.004**
HOMA-IR	3.81(1.12-12.99)	**0.033**
HDL-C	2.28(0.01-358.38)	0.749
ALT	0.98(0.91-1.07)	0.711
UA/Cr	1.44(0.462-4.49)	0.530
Overweight		
LAP	1.10(1.037-1.162)	**0.001**
DBP	0.95(0.827-1.080)	0.406
HOMA-IR	1.33(0.885-1.984)	0.172
TC	3.77(0.053-267.045)	0.541
HDL-C	0.60(0.005-72.349)	0.835
LDL-C	0.408(0.003-48.449)	0.713
ALT	1.01(0.852-1.198)	0.908
AST	1.056(0.757-1.473)	0.747
Obese		
LAP	1.02(0.996-1.050)	0.094
SBP	1.10(0.980-1.225)	0.110
DBP	0.99(0.870-1.123)	0.858
HOMA-IR	1.46(1.100-1.947)	**0.009**
ALT	0.99(0.967-1.012)	0.366
UA/Cr ratio	2.09(1.251-3.479)	**0.005**

SBP, systolic blood pressure; DBP, diastolic blood pressure; HOMA-IR, homeostasis model assessment of insulin resistance; TC, total cholesterol; HDL-C, high-density lipoprotein-cholesterol; LDL-C, low-density lipoprotein-cholesterol; ALT, alanine transferase; AST, aspartate transferase; UA/Cr ratio, uric acid/creatinine ratio. The bold P-value represents a statistical significance.

### The ROC curves of LAP and other biochemical indicators for MS

**Figure 1:**
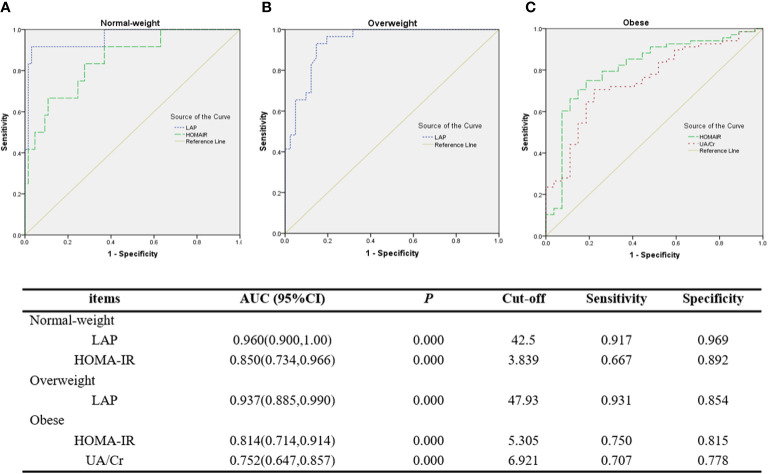
ROC curves of LAP and other discriminators as indicators for MS. **(A)** LAP and HOMA-IR for MS in normal-weight PCOS patients. **(B)** LAP for MS in overweight PCOS patients. **(C)** HOMA-IR and UA/Cr for MS in obese PCOS patients.

ROC curve analysis shows that LAP had an outstanding discrimination index for MS in normal-weight (AUC=0.960, cut-off=42.5) and overweight (AUC=0.937, cut-off=47.93) PCOS patients, with a sensitivity of 0.917/0.931 (normal-weight/overweight) and a specificity of 0.969/0.854 (normal-weight/overweight), respectively. However, HOMA-IR had an excellent discrimination index for MS in normal-weight (AUC=0.850) and obese (AUC=0.814) PCOS patients, with a sensitivity of 0.667/0.750 (normal-weight/obese) and a specificity of 0.892/0.815 (normal-weight/obese), respectively. UA/Cr was found to have a fairly high discrimination index for MS in the obese group, with a sensitivity of 0.707 and a specificity of 0.778, but the AUC value (0.752) was less than that of HOMA-IR (0.814) (**Figure 1**).

## Discussion

The major findings of the present study include the following: (і)a high prevalence (45.04%) of MS among northeastern Chinese women with PCOS; (ii) even in the normal-weight group, a high prevalence (15.58%, almost 15 times as high as that in the control, 1.04%) of MS in PCOS patients was presented. Normal-weight PCOS also showed a significantly higher proportion of dyslipidemia, such as elevated TG and decreased HDL-C levels; (iii) LAP, which is more convenient and economical, showed an outstanding discrimination index for MS in normal-weight and overweight PCOS patients.

Our study presents the overall prevalence of MS among northeastern Chinese women with PCOS as 45.04%, which is quite high compared to a study conducted by Jinxia Zhang et al., who, in their group of 406 women from south-west China with PCOS, reported the prevalence of MS to be 25.62% (NCEP-ATP III criteria) (22). Another large-scale investigation including 833 reproductive-aged PCOS patients from 10 provinces of China reported the prevalence of MS as 19.1% (NCEP-ATP III criteria); however, the BMI-stratified incidence of MS in PCOS was similar to the results presented here (23). The prevalence of MS among women with PCOS reported in other countries ranges from 8.2% to 47.3% (8, 24–26). Different diagnostic criteria used for MS, characteristics and lifestyle factors of the population included, and, in particular, the proportion and severity of obesity alter the prevalence of MS in PCOS patients. A provincial prevalence estimate of adult obesity in China showed a high prevalence of general and abdominal obesity in North women (27). PCOS patients included in the present study showed a high proportion of overweight (28.93%) and obesity (39.26%), and 75.21% of them had abdominal obesity (WC≥80cm). Therefore, the high overall incidence of MS in women with PCOS in north-east China may be due to the high proportion of overweight and obesity, especially abdominal obesity.

Dyslipidemia, which is a common metabolic abnormality in PCOS, has been reported to be associated with increased risk of cardiovascular disease, regardless of BMI (28). The prevalence has been reported at up to 70% in the USA (29) and between 41.3% to 53.1% in the Chinese PCOS population (30). Consistent with the previous studies (22, 30), we found that low HDL-C and high TG levels were the most common abnormalities of MS in PCOS patients. In our study, even normal-weight PCOS patients showed significantly higher rates of elevated TG and decreased HDL-C levels compared to normal-weight controls. Dyslipidemia, which plays an important role in the incidence of MS, may be a possible reason for the high prevalence of MS in non-obese PCOS.

A large proportion of women with PCOS are obese or overweight and exhibit IR (31). Compared with lean PCOS patients of the same phenotype, obese PCOS patients of each phenotype showed higher serum insulin and TG and lower HDL-C significantly (32). Women with PCOS are more likely to have abdominal obesity, which is associated with metabolic diseases, compared to weight-matched controls (33). Abdominal obesity presented not only in obese patients but also in a large proportion of overweight and a minority of normal-weight PCOS patients (34). Of note, a higher prevalence of impaired glucose tolerance and MS were also present in lean PCOS patients than in their BMI-matched controls (35).

Women with PCOS present an adverse reproductive profile, including a high risk of pregnancy-induced hypertension, preeclampsia, and gestational diabetes mellitus, as well as oncological complications, such as endometrial, ovarian, and breast cancers (36). A randomized controlled study conducted in 1508 women with PCOS undergoing *in vitro* fertilization-embryo transfer (IVF-ET) has shown that MS is independently and negatively associated with the cumulative live birth rate (37). In addition, metabolic dysfunction leads to a risk of cardiovascular disease (CVD), which increases with age in women with PCOS (38). Therefore, it is crucial to identify simple, economical, and accurate indicators for early and regular screening for MS in women with PCOS in order to improve IVF outcomes and guide long-term prevention of its adverse consequences.

A series of studies focused on seeking predictive markers of screening MS among the PCOS population, such as lipid accumulation product (LAP), visceral adiposity index (VAI), triglyceride-glucose index (TyG), TG/HDL-C ratio, ApoB/ApoA1 ratio, and so on (8, 14, 39, 40). LAP, a novel index based on a combination of WC and TG, was first introduced to predict cardiovascular risk by Kahn (20). It has been widely reported as a good indicator of MS in the general population, middle-aged and elderly people, and children and adolescents (10, 11, 16). LAP also performs as a superior indicator for detecting MS in women with PCOS from different racial populations compared with other indices (12, 13, 41). However, studies have mostly been performed in the PCOS population as a whole rather than stratified by BMI.

In our study, after multivariable logistic regression and stratification by BMI, LAP was the best indicator of MS in normal-weight and overweight PCOS patients but was not significant in the obese group. The differences in metabolic parameters such as glucose and uric acid, rather than the commonly presented abdominal obesity and dyslipidemia, were more obvious in obese PCOS, which may be the reason why LAP had no significant predictive value for MS in the obese group. Based on the ATP III criteria, we estimated the LAP values of 42.5 and 47.93 as the cut-off points for discriminating normal-weight and overweight PCOS patients with and without MS, respectively. Similar to another study, the diagnosis of MS was based on the updated Chinese Diabetes Society (uCDS) criteria and assessed all the PCOS patients as a whole (13). However, the cutoff point differs from another similar study in which LAP displayed a discrimination index for MS in lean (BMI ≤ 23 kg/m^2^) PCOS patients, with a cut-off value of 23.2 (40). Differences in sample sizes and racial populations being assessed and MS criteria based on may have led to the discrepancy. Compared with obese women with PCOS, non-obese women, especially normal-weight women with PCOS, are more likely to be ignored for their risk of MS. Hence, this study highlights the need for screening a simple and economical predictor in order to implement early intervention methods in clinical practice. Our study indicates that LAP can serve as a reliable marker for assessing MS in non-obese PCOS patients.

Despite these relevant findings, several limitations should be considered in this study. First, our study population was relatively small and single-centered. All of the subjects recruited were infertile and seeking assisted reproductive technology, thus limiting generalization. This may present a possibility of bias in the result. Second, the duration of PCOS was not taken into account, and as a cross-sectional study, it is unable to determine causality. Associations should be further confirmed through longitudinal studies with large sample sizes in the future.

## Conclusions

In conclusion, this study shows that normal-weight and overweight PCOS patients have a significant incidence of MS and should receive as much attention as obese patients. In light of the practical advantages for clinical use, the measurement of LAP might be a useful and convenient tool to predict MS and potential cardiometabolic risks among non-obese PCOS patients.

## Data availability statement

The datasets used and/or analyzed during the current study are available from the corresponding author on reasonable request.

## Ethics statement

The studies involving humans were approved by Ethics Committee of Dalian Women and Children’s Medical Group. The studies were conducted in accordance with the local legislation and institutional requirements. The participants provided their written informed consent to participate in this study.

## Author contributions

WH: Conceptualization, Data curation, Investigation, Writing – original draft, Writing – review & editing. MZ: Funding acquisition, Writing – review & editing. HW: Writing – review & editing. YY: Writing – review & editing. LW: Supervision, Writing – review & editing.
